# Critique of a practice-based pilot study in chiropractic practices in Western Australia

**DOI:** 10.1186/s12998-016-0117-8

**Published:** 2016-10-03

**Authors:** Lyndon G. Amorin-Woods, Gregory F. Parkin-Smith, Lee Nedkoff, Colleen Fisher

**Affiliations:** 1 School of Health Professions (Discipline of Chiropractic) Murdoch University, Perth, Western Australia Australia; 2 Murdoch University Chiropractic Clinic, South Street Campus, 90 South Street, Murdoch, Western Australia 6150 Australia; 3 General Practice and Emergency Medicine Registrar, Busselton, Western Australia 6160 Australia; 4 School of Population Health, Faculty of Medicine, Dentistry and Health Sciences, The University of Western Australia, 35 Stirling Highway (M431), Crawley, Perth, Western Australia 6009 Australia

**Keywords:** Chiropractic, Evaluation study feasibility study, Pilot projects, Professional practice

## Abstract

**Background:**

Practice-based data collection can offer insight into the nature of chiropractic practice and contribute to resolving the conundrum of the chiropractic profession’s role in contemporary healthcare, subsequently informing care service policy. However, there is little formal data available about chiropractic practice to inform decision-makers about the nature and role of chiropractic within the context of a modern multidisciplinary healthcare context in Australia, particularly at a local and regional level.

**Methods:**

This was a mixed-methods data transformation model (qualitative to quantitative) pilot study the purpose of which was to provide a critique of the research design and collect data from a selected sample of chiropractic practices in Western Australia, with a view to offer recommendations related to the design, feasibility and implementation of a future confirmatory study.

**Results:**

A narrative critique of the research methods of this pilot study is offered in this paper covering: (a) practice and patient recruitment, (b) enrollment of patients, (c) data collection methods, (d) acceptability of the study methods, (e) sample size calculations, and (f) design critique.

**Conclusions:**

The result of this critique provides a sensible sample size estimate and recommendations as to the design and implementation of a future confirmatory study. Furthermore, we believe that a confirmatory study is not only feasible, but indeed necessary, with a view to offer meaningful insight into chiropractic practice in Western Australia.

**Trial registration:**

ACTRN12616000434493 Australian New Zealand Clinical Trials Registry (ANZCTR).

Registered 5 April 2016. First participant enrolled 01 July 2014, retrospectively registered.

**Electronic supplementary material:**

The online version of this article (doi:10.1186/s12998-016-0117-8) contains supplementary material, which is available to authorized users.

## Background

The chiropractic profession is well established in many countries [1, 2] yet, even in countries where the profession is formally recognised and regulated, there is often little formal data available about the characteristics of chiropractic practice, practitioners or patients, especially at a local or regional level. Existing data in Australia is either limited or dated, and until recently, there have been no on-going, structured evaluations of chiropractic practice, as found in other healthcare professions such as medicine [3]. A project has recently commenced in Australia collecting data from chiropractic practices, being the ACORN project [4], however but this project is still evolving, with initial data to be presented mid-2016.

Existing data on chiropractic practice or patients are primarily from Europe, the USA and Canada, with few studies of satisfactory quality providing insight into chiropractic practice in Australia. The amalgamated findings from these studies [5–18] indicate that the majority of patients consult chiropractors for spinal pain, mainly lower back, and to a lesser extent neck and related neuro-musculoskeletal disorders. Several Australian studies have reported on samples collected in the state of Victoria, with the exceptions being Ebrall [5], Xue [13] and Brown [17] who reported on national samples.

The healthcare demand issues facing developed countries are now well-known, particularly related to the needs of an aging population, chronic disease, often limited workforce and dramatic cost inflation [19]. Therefore, the emerging focus in healthcare is to have care services delivered using mechanisms or strategies that are cost-efficient and evidence-based, while engaging with the broadest applicable workforce [20]. In Australia, as in other countries, government-generated models of care for musculoskeletal health care provision, which includes spinal pain, have increasingly focused on shifting services from hospital to community-based settings, and improving access to multidisciplinary care. This shift is aimed at improving outcomes for patients, containing healthcare costs, and making use of the available, appropriately trained workforce [21, 22]. There are thus opportunities for the chiropractic profession to play a role in the context of a multi-disciplinary care, and particularly in the area of musculoskeletal/spinal health.

Practice-based data collection offers information and insight into the characteristics of practitioners and patients and thus may contribute to resolving the conundrum of chiropractic’s role in contemporary healthcare [23–25]. These data are also fundamental to facilitating interaction and understanding between educators, professional representative bodies, clinicians, consumers and insurers, alongside other stakeholders, concerning the nature and role of chiropractic within the milieu of modern multidisciplinary healthcare [26, 27]. This study addressed gaps in knowledge by gathering local (WA) data in a practice-based setting using electronic means since this method is known to be cost effective [28].

### Aims

The primary purpose of this paper is to provide a critique of the research design and methods of the pilot study and to offer recommendations related to the design, feasibility and implementation of a larger definitive study. The topics for the narrative critique of the research methods of this pilot study are: (a) practice and patient recruitment, (b) enrollment of patients, (c) data collection methods, (d) acceptability of the study methods, (e) sample size calculations, and (f) design critique. The insights and recommendations generated from this type of study are intended to inform future studies [29].

Although a summary of the methods and research process is given in this paper, the detailed findings relating to the practices and people consulting chiropractors in the study are published elsewhere.

## Methods

**Fig. 1:**
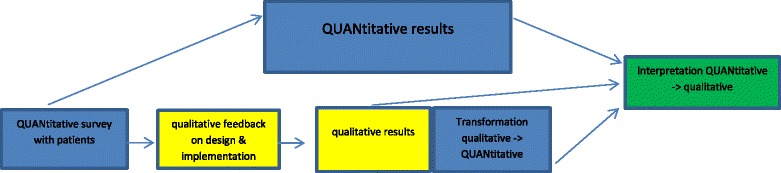
The mixed methods design of the PIStAChiO study

We have previously published a full, detailed account of the pilot study design and data collection (including the questionnaires and instruments used) [30] (Fig. 1).

**Fig. 2:**
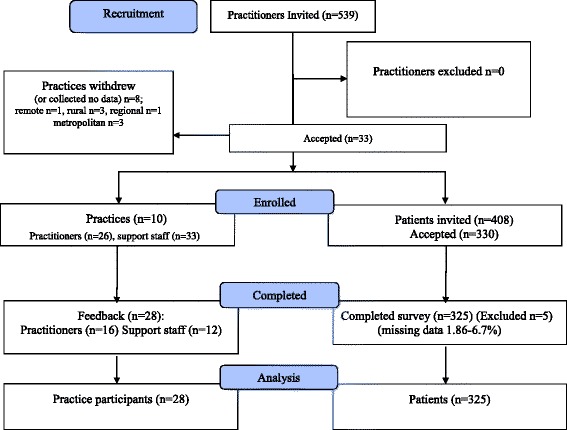
Flow Diagram PIStAChiO

A non-random sample of independent private chiropractic practices was recruited between July-September 2014 by email and at conferences and meetings. Responding practitioners and support staff were instructed in the study protocols either in person (*n* = 8 metro, 1 regional) in conjunction with email or by phone (*n* = 4 metro, 1 regional, 3 rural, 1 very remote). Consecutive adult new patients self-presenting to these practices being invited to participate by support staff when they filled out intake documentation. Data for analysis were collected using a computer-based online questionnaire obtaining various patient demographic data and human quality of life measures. Both qualitative and quantitative responses were also collected from the practice practitioners and support staff. Data were collected online via an internet portal, in the practice, on new patients between July and September 2014 (Fig. 2).

## Results

### Recruitment of practices

Initially, a sample of 18 chiropractic practices was recruited, however 8 of the original 18 practices either withdrew or failed to collect data – this represented a 44 % drop-out. Some withdrew citing a perceived interruption to patient flow through their clinics, some described unforeseen staff and administrative problems, and others gave no reason for withdrawal. The consequence of the practice drop-outs was a significant reduction in the number of regional, rural and remote practices participating in the study, and therefore, less data available from these geographical regions. In addition, some of the participating practices collected data for less than the full 12 weeks of the study.

### Patient recruitment and enrollment

The recruited practices (metropolitan *n* = 8, regional *n* = 2) had an overall strong positive new patient response rate of 81.7 % with subsequent sample sizes of 301 patients from metropolitan practices and 24 patients from regional practices. One regional practice incorrectly collected data from five patients under the age 18, thus these responses were excluded from the data set before analysis.

## Discussion, critique and recommendations

### Recruitment of practices

For future studies a possible mechanism to mitigate the drop-outs is to secure continuing professional development (CPD) credits for participating practitioners – this may motivate participation. Also, it would be important to engage with, and orient practitioner’s in-person, preferably in situ since none of those inducted in person dropped out. Practical constraints limited in-person contact in this study, so we feel that direct engagement in future studies would establish a partnership, expose problems, or reveal study implementation challenges, thereby reducing the chance of drop-out. One method of enhancing recruitment and reducing drop-out rates in the past has been through the presentation and demonstration of the study and participation invitation at a conference / regional meeting of chiropractors. This has apparently previously worked well in Sweden [6]. Future studies would also likely benefit from using a formal Practice-Based Research Network (PBRN) ‘community’ of practices where participants may interact and share common experiences and facilitate collegiality, thus maintaining participation. We also found where a group of chiropractors in one practice decide to take part it is possible that commitment and “collegiality” reduces the risk of drop-out despite the mild/moderate disruption to daily practice when taking part in research.

#### Recommendation

Although the initial response was encouraging, future studies will need to plan for a significant proportion of drop-outs. Continuous Professional Development (CPD) credit for participation, collegiality and personal engagement should facilitate participation and reduce drop-outs.

### Patient recruitment and enrollment

The most salient factor with respect to patient recruitment was’*support staff fatigue*, where there was a noteworthy increase in patients ‘declining’ as the study progressed over time. The authors speculate that practice support staffs’ motivation and enthusiasm diminished over the duration of the study, with consistent generic excuses being listed as ‘*didn’t want to*’ and ‘*no time*’. Thus, the recommendations for the conduct of future studies that require strong ‘buy in’ from support staff where: (a) the study be conducted over a shorter period of time with a smaller number of patients being recruited per practice, (b) broaden the recruitment of practices, and (c) the use of creative ways to reward support staff for their efforts. For example; financial remuneration, or (paid) time off from clinic duties. There are of course implications both ethical and practical for any study and for the participating chiropractic clinic with provision of incentives.

#### Recommendation

Incentive or rewards for chiropractic practices and their staff to participate in a study may encourage participation and maintain motivation. A future study would also be more feasible if a broad sample of chiropractic practices were recruited from across regional/geographical areas.

### Consent and patient information sheets

Consent was successfully obtained from all participants however feedback from practice staff was that consent and information sheets were too cumbersome. Given institutional ethical (HREC) requirements, these documents were necessary, but could be condensed by using drop-down option boxes or pop-up information boxes, as commonly used in contemporary online surveys.

#### Recommendation

The length and detail of information and consent forms was a barrier to participation. These should be revised and shortened wherever possible, using plain language.

### Anonymity, confidentiality and data management

Participant records and questionnaires were made anonymous and data could not be identified for individual patients. Thus, there were no issues with respect to patient privacy or confidentiality. Practitioner bias was reduced by having the patients complete the questionnaires prior to their consultation.

### Questionnaire design and data collection

The online questionnaire used in this study (Additional file 1) was based on those used in similar published studies, covering numerous patient characteristics, demographics, and also quality of life measures not previously included in published studies [18]. In addition, the online questionnaire was trialed with the principal chiropractor at each recruited practice using a test version of the internet portal. The feedback from these practitioners was used to refine the questionnaire.

A full critique of the questionnaire is also provided in Additional file 1. The main insights drawn from evaluation of the questionnaire were that: (a) questionnaires should be checked for correct completion, since missing data and errors could be avoided with better supervision or checks, (b) there should be a sufficient number of answer options related to each question. For example, there should be a drop-down box with sufficient options for the selection of the patient’s occupation, and (c) the Quality of Life questionnaire(s) should be presented in a clear format with simple language, so to facilitate accurate completion and to avoid questions being omitted.

In terms of chiropractic-related research, the use of the online survey portal (via tablet computer) and questionnaire was novel - past surveys/questionnaires have all been paper-based. The online portal was considered accessible, quick and had the functionality to rapidly collate and analyse collected data. There were some reported instances of patients declining to participate or difficulties specifically because of the technology, or aversion to the technology. However, on the whole, this study showed that the online method of data collection was accessible and feasible.

#### Recommendation

To spread the workload of data collection and reduce the time taken for patients to complete questionnaires, we recommend that practitioners (clinicians) collect the clinical data while support staff gather financial and demographic data, thus freeing patients to provide only patient-specific data, thereby avoiding onerous questionnaire completion time with consequent decreased likelihood of missing data and dropouts by patients. This recommendation thus still addresses potential practitioner bias while addressing other issues we identified.

### Online survey tool

A well-known internet-based survey portal (SurveyMonkey™) was used for its convenience and since the primary researcher already had some experience with this platform [31]. There are an increasing number of platforms available with an ever expanding technical capacity for complex design including skip logic, optional question formats and pop up instructions [32]. For example the REDCap Consortium composed of 1,318 active institutional partners (including UWA) in 88 countries is a mature, secure web application for building and managing online surveys and databases [33]. Another viable alternative is that offered by *Research Australia*. This site was developed in WA and has been used by the Fremantle Hospital and Pain Service to allow patients to complete health related questionnaires given by their health practitioner, from one convenient online location, the aim of which is to simplify the collection of key information [34]. The advantage of this tool is that a variety of outcome measures and HQoL tools are electronically formatted and licensed for research and clinical use.

Online surveys are becoming an almost essential research tool of modern research, with capabilities that offer a wide range of textual options, format control and graphics sophistication, links and menus. These capabilities enable quick referencing, text entries, help avoid omissions [35, 36]. In the modern world, online surveys may be preferred even though privacy is a minor concern to some participants [37, 38].

This study had response rate of >81 % with very little missing data. This finding supports the usefulness of online surveys; being faster, simpler and cheaper, while significantly improving efficiency and accuracy [36]. Overall the online portal and questionnaire used in this study was satisfactory, although quite limited with respect to visual design, and the process of using skip logic requires quite an advanced degree of familiarity with the platform. The platform did not provide the ability to code variables and ‘clean’ raw data for download in statistical software format which necessitated subsequent relabeling and refining. Merging data from the various practice portals prior to download would also be a useful feature for future studies.

#### Recommendation

Online, electronic data collection is highly recommended, but the software platform needs to be carefully chosen so that data management/analysis is efficient.

### Acceptability of the study methods

This study had a 44 % dropout of chiropractic practices after initially agreeing to participate. Some withdrew citing a perceived interruption to patient flow through their clinics. However, of those practices that did complete the study, 50 % indicated they would be open to participating in future studies, while the other 50 % were neutral. It is important to report that support staff overall were less likely to want to participate in future research. The main negative observation received from support staff was the length of time taken to complete the survey and the subsequent disruption to normal clinic flow. Since this study relied heavily on the support staff for implementation, this will need to be addressed in future studies.

#### Recommendation

Explicit recognition of staff efforts and possibly non-coercive incentives would reward staff and motivate them to participate in further or future studies.

### Research design critique

Mixed methods research (MMR) provides many strengths that offset the weaknesses of either quantitative or qualitative designs, the key strengths being: (a) the voices of participants are directly ‘heard’ and recorded in qualitative research, (b) all of the potential tools of data collection are all available to the researcher when both methods are used, and (c) the researcher is enabled to utilise all the various types of data available to answer the research question [39, 40]. The philosophical basis of this work was pragmatism following the description of this worldview as articulated by Morgan [41]. Pragmatism, with its focus on ‘what works’ allows the researcher to move beyond philosophical questions about mixing or combining methods and allows for an integrated methodology for the health and social sciences [41]. Green (2001) persuasively makes the observation that if we want more evidence-based practice, we first need more practice-based evidence [42, 43] with a view offer generalisable solutions to clinical problems and improvement of care delivery systems.

#### Recommendation

Mixed-methods research is recommended to obtain meaningful and rich insights into both patients and practices. However, the breadth and depth of data collection should be balanced with the administrative and workload demands of such methods and data collection.

### Sample size estimation for a confirmatory study

The recommended sample size is the best-estimate of a sample to ensure a reasonably powered, yet feasible, confirmatory study. Values used in calculations are based on available data, such as population size of WA, the estimated WA population with spinal pain, and the best-estimate values for the intra-class correlation co-efficient, effect sizes and confidence intervals.

According to the latest data at the time of the study, WA had a population of 2,565,600 people, with about one fifth (20 %) under the age of 18 years, implying the WA adult population was approximately 2,052,480 people [44]. Point prevalence of spinal pain is approximately 15 % of the population, based on published data [45–48]. This suggests that the point-prevalence of spinal pain in the WA adult population would be around 307,872 people. Approximately 10 % of adults with spinal pain seek care [46, 49] suggesting a point-prevalence population of about 30,787 people seeking care for their spinal pain in WA. The estimated population with spinal pain that seek care who then consult a chiropractor is around 12–16 %, [13, 46, 50] indicating the number of the adult population with spinal pain that seek care and consult a chiropractor in WA is approximately 3694 people.

Regarding the sample size calculation using data sets from this pilot study, the “best-fit” statistical test was used, this test being the difference between two independent means, calculated using a two-tailed test, where an effect size is determined using the means and standard deviations for the respective data sets. Although this approach is not ideal for a cross-sectional study, the results did provide useful insights into a required sample size for a future confirmatory study. An estimate of the cluster effects and variance inflation factor (VIF), as encountered in controlled clinical trials involving multiple sites/practices, was also performed with a view to determine the potential influence of these factors on sample size. Latterly, a compromise VIF was calculated and an estimated sample size offered for a larger study.

Using select data sets from the outcome measures, being the Physical Component Score (PCS) & the Mental Component Score (MCS) of the SF-12 (Study PCS mean 47.9, Societal Norm PCS mean 49.63; Study MCS mean 37.38, Societal Norm MCS, mean 49.37; Study PIQ mean 56.12, Societal Norm PIQ mean 50.0) and the cumulative pain score from the PIQ-6, a sample size estimation was calculated for each data set when comparing them to published societal norms data [51]. G*Power statistical software [52] was utilised where the difference between two independent means was calculated using a two-tailed test where α = 0.05, 1-β = 0.9, and the lowest effect size obtained across the data sets was 0.209, generating a calculated total sample size of 964 total, or 241 per region over 4 geographical regions (if the study is to include data from various geographical regions and so to enable comparison). With 10 % dropout, these values would need to be inflated to 1060 and 265, respectively.

Overall, a reasonable and feasible sample size would be a compromise between sample size estimates, being: 1643 (generic calculation), 1828 (specific calculation using study data), and 1334 (as used in a published trial on spinal pain [53]), respectively. Thus, the total recommended sample size offered by the authors of this pilot study for a future larger trial is around 1600 (90 % CI 1181 to 2023; 95 % CI 982 to 2222), or approximately 400 per region over four regions (metropolitan, regional, rural and remote).

Offering recommendations and an estimation of the practice sample size is more difficult to determine. The default position is to have as many practices involved as possible from as many regions as possible. However, there needs to be a strategy to facilitate the involvements of an appropriate number of practices across the four regions to compensate for practice drop-out and the assumed lesser number practices in rural and remote geographical settings. In this pilot study, 18 practices expressed an interest in participating, yet eight dropped out prior to commencement, leaving ten practices to participate – this represents an overall 44 % dropout of practices. We observed that data from an average of 32 patients was collected at each of the ten participating practices in this study. From the estimation of required sample size for a larger trial of 400 participants is needed per geographical region. The number of practices per region therefore would be calculated as (400/32) + (44 % dropout × (400/32)) = 18 practices per region. Therefore, approximately 18 practices per region are recommended for a larger study (total of 72 practices).

#### Recommendation

Sample size estimates offered in this section are merely to provide an impression of samples needed for a confirmatory study. The best estimations for sample size and practice numbers for a future larger chiropractic cross-sectional study would be around 400 participants per geographical region (total 1600 patient participants), with data collected from approximately 18 practices per region (total of 72 practices). However, as stated in the sample size estimation using the traditional calculation for cross-sectional studies, a future study may be able to be done with around 960 participants, within the context of a well-designed, robust study. Research budgets and design should consider these estimations in study planning, although funding and practical constraints may limited sample sizes in future studies.

### Strengths and limitations

This was an innovative mixed methods pilot study, being a first for chiropractic research in the state of Western Australia, which set out to assess the feasibility and applicability of collecting data in a practice-based setting using electronic technology. This study provided insights and directions in both quantitative and qualitative domains while demonstrating the feasibility of electronic data capture in chiropractic practices in WA. The metropolitan practices provided sufficient data to reach the pilot target sample size with a very low level of missing data. The target of recruiting a mix of 80–20 % metropolitan to non-metropolitan practices was partially achieved, however the lack of rural and remote practices means no conclusions can be drawn for those regions. The patient response rate for the practices that collected data was very strong, providing adequate data quality and breadth for meaningful analysis. It is worth noting our study collected data on more *new* patients than the two comparative preceding published works [5, 18]. Collecting data from only new patients means there was no statistical correction necessary to avoid ‘double counting’ as was the case, for example, in the COAST study where 13 % of encounters were repeat visits [18]. The relative ease with which the study was designed, set up, and implemented while providing meaningful direction for future works ensures the study could be easily replicated and expanded.

There were, however, limitations to this study. There was a lack of data collected from rural and remote locations despite several practices initially being recruited. Therefore we were unable to compare data from geographical regions due to insufficient data. We have noted certain domains were not fully addressed in the questionnaire, including duration of complaint, which would have allowed stratification according to acute, sub-acute and chronic subsets. Since we recruited only practices with the ability to provide internet access, we are not able to draw conclusions regarding those practices that are still entirely paper-based. Finally, we collected data only from adults, future studies would need to include people under the age of 18.

## Conclusions

The narrative critique of this pilot study suggests that a future confirmatory is feasible and, indeed, necessary, with a view to obtain insights into chiropractic practice and the potential role of chiropractic within an evolving contemporary healthcare system in Western Australia. Reasonable sample size estimates and design recommendation are provided to inform the design, planning and budgeting for future studies.

## Abbreviations

(C)SMT, (Chiropractic) spinal manipulative therapy; HQoL, human quality of life (measure); MCS, mental component score; PCS, physical component score; PIQ, pain impact questionnaire; PISTACHIO, practice-based investigation and study of attendees at chiropractic offices; SF, short form (12 & 36); VIF, variance inflation factor

## Additional file

Additional file 1: Questionnaire and detailed critique of questionnaire. (DOCX 22 kb)
