# Supplementary material for: Critique of a practice-based pilot study in chiropractic practices in Western Australia
Source: Chiropr Man Therap. 2016 Oct 3;24:38. doi: 10.1186/s12998-016-0117-8 (PMC5046802; doi:10.1186/s12998-016-0117-8)
Supplement: Additional file 1: — Questionnaire and detailed critique of questionnaire. (DOCX 22 kb) [file 12998_2016_117_MOESM1_ESM.docx]

**Additional file 1**

1. *Online Questionnaire*

Patient Demographics, Reason for Presentation and Lifestyle

1. What is your gender?

2. What is your age?

3. What language do you mainly speak at home?

4. Do you identify as an Aboriginal or Torres- Strait Islander person?

5. Which of the following best describes your current occupation?

Architecture and Engineering

Arts, Design, Entertainment, Sports, and Media

Business and Financial Operations

Cleaning and Maintenance

Community and Social Service

Computer and Mathematical

Construction

Education, Training, and Library

Farming, Fishing, and Forestry

Food Preparation and Serving Related

Other (please specify)

6. Which best describes the source of payment for your visit?

Department of Veterans’ Affairs (DVA)

Insurance Commission (ICWA) or Transport Accident Commission

Medicare

Patient paid 100% [No insurance]

Patient paid partial [Co-payment]

Private health insurance

No charge

Workers Compensation

Other (please specify)

7. How did you find out about this clinic?

Word of mouth (personal referral)

Referral from a medical practitioner

Referral from another health practitioner

Referral from another source (eg; Lawyer)

Friends or family

Print advertisement

Internet

Social Media

Signage

Other (please specify)

8. Which of the following best describes the MAIN reason you have consulted this chiropractor?

Low Back problem

Mid Back (between the shoulders) problem

Neck problem

Muscle problem

Health maintenance or preventive care

Back syndrome with radiating pain

Musculoskeletal symptom or complaint

Headache

Sprain or strain of joint

Shoulder problem

Nerve-related problem

General symptom or complaint

Bursitis, tendinitis or synovitis

Kyphosis and scoliosis

Foot or toe symptom or complaint

Ankle problem

Osteoarthrosis, other (not spine)

Hip symptom or complaint

Leg or thigh symptom or complaint

Musculoskeletal injury

Depression

Other (please specify)

9. Which of the following are reasons OTHER than the main reason you have consulted this

chiropractor? (same options as above)

10. Have you been diagnosed by a medical practitioner (doctor) with any other health conditions before you have consulted this chiropractor?

Cardiovascular problem

Cancer

Diabetes

Respiratory problem

Neurological disorder

Depression or mental illness

Other (please specify)

11. Do you smoke?

12. In a typical week how many alcoholic drinks would you consume?

13. Medication and nutritional supplements

Please indicate how many different types you take per day

Prescription medication

Over the counter medications

Nutritional supplements

14. Have you consulted any other health practitioners for the main complaint you are seeing the chiropractor today?

Medical Practitioner

Physiotherapist

Osteopath

Massage Therapist

Occupational Therapist

Acupuncturist

Other (please specify)

15. Have you been to a chiropractor before this one?

1. *Critique and Recommendations of the Online Questionnaire*

A narrative critique and recommendations of the online questionnaire is offered in this appendix. The purpose is to provide the reader with constructive advice as to the design of a future questionnaire. Each question from the online questionnaire is stated, followed by commentary and a recommendation(s).

1. “Please enter the unique codes given to you by the clinic staff.”

Whilst this would seem a straightforward request, some patients incorrectly entered clinic codes or various wrong numbers in these fields. This did not affect the data as they were automatically sequentially coded by the software.

Recommendation: Coding or such domains could be omitted completely in a future study. However, if coding is used, then the clinic staff should enter these to avoid errors.

2. “What is your gender?”

The gender domain has historically been a simple case of two options, male or female. Since a High Court judgment in Australia in April 2014, persons who have undergone sexual reassignment surgery have now won the right to describe themselves as neither male nor female [1]. The High Court made clear indications in their judgment that people of non-binary gender identities are in the community and they need to be recognized legally.

Recommendation: A third option, labeled ‘non-specific’, should be included in a questionnaire. However, reporting of these data has to be balanced against any potential for breach of privacy since The Privacy Amendment (Enhancing Privacy Protection) Act 2012 [2], (which amended the Privacy Act 1988) makes strong provision for the protection of an individuals’ identity in this context.

3. “What is your age in years?”

Age was initially proposed to be collected as a stratified discrete variable, however to allow more sophisticated analysis the decision was made to collect age as a continuous variable.

Recommendation: Collect age as a continuous variable.

4. “What language do you mainly speak at home?”

Language categories were chosen to reflect the languages spoken at home in WA according to the Australian Bureau of Statistics 2011 Census of Population and Housing [3]. These languages are in descending order of frequency (after English); Italian, Mandarin, Cantonese, Vietnamese, Filipino/Tagalog, Arabic, Afrikaans, Australian Indigenous Languages, Indonesian and German. There were a number of patients who responded ‘other’ since the list provided did not include their language including; Bengali, French, Gujarati, Hindi, Japanese, Portuguese, Spanish, Swedish, Tamil and Thai.

Recommendation: If collecting and analyzing language-specific data or related socio-demographic factors was important, then a comprehensive list should be made available from which a participant could choose.

5. “Do you identify as an Aboriginal or Torres- Strait Islander person?”

Aboriginal and Torres Strait Islander people are under-identified in many health-related data collections. This is likely to also be true for Indigenous peoples in other countries. Self-report in response to the standard Indigenous status question is considered the most accurate means of ascertaining a person’s Indigenous or non-Indigenous status [4, 5] thus, Indigenous identification in PIStAChiO was collected according to the protocols set out in the national best practice guidelines for collecting Indigenous status in health data sets [6].

Recommendation: Four standard response options should be provided to patients to answer the question in two parts; (1) ‘Yes/No/prefer not to answer’; (2) (a) Aboriginal but not Torres Strait Islander origin, (b) Torres Strait Islander but not Aboriginal origin, (c) both Aboriginal and Torres Strait Islander origin. That said, optimal collection of data regarding Indigenous identification has always been a challenge.

6. “Which of the following best describes your current occupation?”

There were some issues related to the list of occupations evidenced by the number of patients who responded ‘other’ since the list provided did not adequately describe their occupation. Some of these occupations for example were; compliance officer, designer, education assistant, educator, farmer, and so forth. In addition the list provided contained some categories that overlapped and, thus, these were aggregated for analysis (e.g. managers and professionals).

Recommendation: A simpler more inclusive and standardized occupation list should be framed for future studies. For example, selection from a comprehensive drop-down box in the software.

7. “Which best describes the source of payment for your visit?”

As in the previous domain, the source of payment of the visit reflected previous survey results with both these domains being listed alphabetically. At first glance there appeared contradictory responses with respect to the numbers who reported Medicare as being a source of payment. There was apparently confusion between ‘Medicare’, the government's national healthcare service, and ‘Medibank Private’ a large private health insurer, since 52% who selected ‘Medicare’ also indicated co-payment or private insurance as a source of payment. This would need to be clarified in future studies, ideally through payment records. In addition there appeared to be some confusion between patient payment and private insurance options, thus these data were aggregated for analysis.

Recommendation: If collecting financial information was important in a future study, there would need to be clearer options regarding payment data. In fact these data would be better collected from financial patient record data, as opposed to being patient self-reported. This however, may present logistical challenges that would likely need ethics approval.

8. “How did you find out about this clinic?”

Various options, such as; ‘word of mouth’ and ‘friends or family’, and ‘internet’ and ‘social media’ overlap, thus one domain for each of these would have been sufficient.

Recommendation: These options were considered adequate to capture the data however fewer options may avoid confusion.

9. Questions 9 and 10 are considered together. “Which of the following best describes the main reason you have consulted this chiropractor?”

10. “Which of the following are reasons other than the main reason you have consulted this chiropractor?”

A number of the options were rarely selected the list on offer was likely too long to be relevant to the patient. This question should also have been accompanied with other important clinically relevant domains such as causes and aggravating factors linked to the patient's presenting complaint. In particular, duration of complaint would be recommended to be able to stratify according to acute, sub-acute and chronic subsets.

Recommendation: An abbreviated, more intuitive list of presenting complaints would be sufficient, along with the inclusion of duration of complaint, causes and aggravating factors domains.

11. Have you been diagnosed by a medical practitioner (doctor) with any other health conditions before coming to this chiropractic clinic?

This question was framed according to the leading causes of mortality and morbidity in the Australian population listed in order of incidence by the Australian Institute of Health and Welfare [7]. It is likely, however, that patients under-reported their co-morbidity status, as the prevalence of diabetes and cardiovascular disease as reported in this study was lower than reported in the general population. The issue of co-morbid conditions is very broad - for example, people may know they have had a heart attack but not realize that they have ‘cardiovascular disease’, similarly for someone with asthma and respiratory disease. There are varying degrees of accuracy for self-reporting of conditions - people generally self-report accurately on conditions such as heart attack (myocardial infarction) but are less reliable on heart failure, hypertension and diabetes [8, 9].

Recommendation: Cross matching with practitioner reported data may address this gap in future.

12. Question 12 and 13 are considered together. “Do you smoke?” (“Yes”,” No”,” I used to but gave up”)

13. “In a typical week how many alcoholic drinks would you consume?”

Smoking status was collected by present behaviour in addition to previous habit, and alcohol consumption levels were based on guidelines of safe consumption as published by WA Health. The question, however, should have been framed to also ask about patterns of alcohol consumption (i.e. >2 standard drinks per day) in addition to overall levels.

Recommendation: Exploring smoking consumptions is important due to their indirect link to non-malignant back pain. Furthermore, if subgroup statistical analysis is anticipated in a future study, with a view to explore the association between presenting complaint(s) and lifestyle factors, then sufficient data needs to be collected regarding each factor (Variable).

An example patient options and how data regarding smoking and alcohol consumption may be collected:

• Do you smoke? Smoker, Ex-Smoker, Never Smoked

• Frequency? Daily, Weekly, Less than weekly

• Amount: (insert number)

• Year commenced? Number of years? (number of pack years automatically calculated)

• Ever tried to quit? If so, how long was the longest period of abstinence?

• Days of the week you consume alcohol? Every day, 5-6 day per week, 4-3 days per week, 1-2 days per week, 1-2 days per month, less than monthly, never (non-drinker)

• On a day you drink alcohol, how many standard drinks do you have? (insert number)

• Do you binge drink (more than 6 standard drinks in one occasion)? Daily or almost daily, Weekly, Monthly, Less than monthly, Never

• Are you concerned about drinking? Yes/No/Don’t know

14. Medication and nutritional supplement. Please indicate how many different types you take per day”

Dosages per day for prescription medication, over the counter medications and nutritional supplements were collected.

Recommendation: Fewer options may have been sufficient for the purposes of a future study.

15. “Have you consulted any other health practitioners for the main complaint you are seeing the chiropractor today?”

The list of options for previous practitioners was based on the practitioners most often utilised in Australia for back pain [10].

No further recommendations offered.

16. “Have you been to a chiropractor before this one?” (for items 11-16)

These questions appear to have been adequately framed for the purpose of this study.

Recommendation: A future study may benefit from cross matching of data. Questions 15 and 16, on reflection, may have been better situated following Question 11. Question 11 would also be better asked of a clinician following a health history.

17. Human Quality of Life Measures

The only missing data in the entire survey were found in these domains, presumably since not all parts of all questions could be made required fields due to the technical limitations of the survey platform. Some patients (n=5, 1.5%) failed to answer any questions in the SF-12 and PIQ, and missing data in various domains of both HQoL’s ranged from 1.8-6.7%. Given feedback on the length of the survey from support staff and practitioners, it would probably have been sufficient to have only administered the SF-12, since it gathered data on pain levels in addition to overall well-being and mental health components. As future studies would be expected to examine follow-up and outcomes of management, these data may prove useful as baseline measures of the patients seeking chiropractic care.

Recommendation: The SF-12 HQoL is an appropriate measure to administer alone in this context. Future studies with a particular focus on quality of life measures would benefit from a more in-depth evaluation, particularity as an outcome measure. Overall, the HQoL seem to be very useful and appropriate outcome measure, feasible and valid to be used in future studies.

References

1. **NSW Registrar of Births, Deaths and Marriages v. Norrie**, *High Court of Australia*; 2014 April.

2. **Privacy Amendment (Enhancing Privacy Protection) Act.** Canberra: *Office of the Australian Information Commissioner*, Commonwealth of Australia; 2012.

3. ABS: **Census of Population and Housing Usual Residence Data**. Canberra: Commonwealth of Australia; 2011.

4. **What details do we know about the Indigenous population?** [http://www.healthinfonet.ecu.edu.au/health-facts/health-faqs/aboriginal-population]

5. AIHW: **Towards better Indigenous health data,** Cat. no. IHW 932013.

6. AIHW.: **National best practice guidelines for collecting Indigenous status in health data sets**. *vol. Cat. no. IHW 29*. Canberra: Australian Institute of Health and Welfare; 2010.

7. AIHW: **Australia's health 2010**. Cat. no. AUS 122. Canberra: *Australian Institute of Health and Welfare*; 2010.

8. Okura Y, Urban LH, Mahoney DW, Jacobsen SJ, Rodeheffer RJ: **Agreement between self-report questionnaires and medical record data was substantial for diabetes, hypertension, myocardial infarction and stroke but not for heart failure**. *J Clin Epidemiol* 2004, 57(10):1096-1103.

9. Tisnado DM, Adams JL, Liu H, Damberg CL, Chen WP, Hu FA, Carlisle DM, Mangione CM, Kahn KL: **What is the concordance between the medical record and patient self-report as data sources for ambulatory care?** *Med Care* 2006, 44(2):132-140.

10. Walker BF, Muller R, Grant WD: **Low back pain in Australian adults. Health provider utilization and care seeking.** *J Manip Physiol Ther* 2004, 27(5):327 - 335.
